# The Post-Translational Modification Networking in WNK-Centric Hypertension Regulation and Electrolyte Homeostasis

**DOI:** 10.3390/biomedicines10092169

**Published:** 2022-09-02

**Authors:** Shiuan-Chen Lin, Chun Ma, Kao-Jung Chang, Han-Ping Cheong, Ming-Cheng Lee, Yuan-Tzu Lan, Chien-Ying Wang, Shih-Hwa Chiou, Teh-Ia Huo, Tsui-Kang Hsu, Ping-Hsing Tsai, Yi-Ping Yang

**Affiliations:** 1 School of Medicine, National Yang Ming Chiao Tung University, Taipei 112304, Taiwan; 2 Department of Medical Research, Taipei Veterans General Hospital, Taipei 11217, Taiwan; 3 School of Medicine, National Taiwan University, Taipei 10617, Taiwan; 4 Institute of Clinical Medicine, National Yang Ming Chiao Tung University, Taipei 11221, Taiwan; 5 Institute of Pharmacology, National Yang Ming Chiao Tung University, Taipei 11221, Taiwan; 6 Department of Medicine, Cheng Hsin General Hospital, Taipei 11220, Taiwan; 7 Division of Infectious Diseases, Department of Internal Medicine, Cheng-Hsin General Hospital, Taipei 11220, Taiwan; 8 Division of Colon & Rectal Surgery, Department of Surgery, Taipei Veterans General Hospital, Taipei 11217, Taiwan; 9 Department of Critical Care Medicine, Taipei Veterans General Hospital, Taipei 11217, Taiwan; 10 Division of Trauma, Department of Emergency Medicine, Taipei Veterans General Hospital, Taipei 11217, Taiwan; 11 Department of Physical Education and Health, University of Taipei, New Taipei 10478, Taiwan; 12 Department of Ophthalmology, Taipei Veterans General Hospital, Taipei 11217, Taiwan; 13 Genomic Research Center, Academia Sinica, Taipei 11529, Taiwan; 14 Department of Ophthalmology, Cheng Hsin General Hospital, Taipei 11220, Taiwan; 15 Institute of Food Safety and Health Risk Assessment, School of Pharmaceutical Sciences, National Yang Ming Chiao Tung University, Taipei 112304, Taiwan

**Keywords:** WNK kinase, post-translational modifications, blood pressure regulation, NCC, NKCCs, ROMK, ENaC, SPAK/OSR1, UMOD, PHAII

## Abstract

The with-no-lysine (WNK) kinase family, comprising four serine-threonine protein kinases (WNK1-4), were first linked to hypertension due to their mutations in association with pseudohypoaldosteronism type II (PHAII). WNK kinases regulate crucial blood pressure regulators, SPAK/OSR1, to mediate the post-translational modifications (PTMs) of their downstream ion channel substrates, such as sodium chloride co-transporter (NCC), epithelial sodium chloride (ENaC), renal outer medullary potassium channel (ROMK), and Na/K/2Cl co-transporters (NKCCs). In this review, we summarize the molecular pathways dysregulating the WNKs and their downstream target renal ion transporters. We summarize each of the genetic variants of WNK kinases and the small molecule inhibitors that have been discovered to regulate blood pressure via WNK-triggered PTM cascades.

## 1. Introduction

Electrolyte homeostasis plays a pivotal role in blood pressure regulation [1]. Regarding the genetic etiologies of blood pressure dysregulation, renal channelopathy represents a group of disorders with distinct mutational profiles that have also instigated the generation of different transgenic mouse models [2]. Due to the recent advances in molecular diagnosis, the identification of mutations in ion channels and upstream regulator kinases are emerging in clinical scenarios of familial blood pressure disorders, such as Bartter’s Syndrome, Gitelman Syndrome, Liddle’s Syndrome, and pseudohypoaldosteronism type II (PHAII) [3,4,5,6], which stem from WNK-dependent channelopathies. WNK kinases are the central kinase family in the regulation of ion channel activity implicated in blood pressure regulation. Mechanistically, WNK kinases regulate the activity of renal cation-chloride transporters by activating the SGK1-WNK-SPAK/OSR1 phosphorylation cascade, which is responsible for the post-translational modifications (PTMs) of ion channels in renal tubules, thus modulating ion transport efficiency.

Aberrant renal SGK1/WNK-SPAK/OSR1 regulation is involved in disease conditions such as Gordon’s Syndrome and Gitelman-like conditions [7,8,9,10]. Upstream kinases of the WNK-SPAK/OSR1 axis, such as SGK1 (Serum/Glucocorticoid Regulated Kinase 1) and protein kinase B (also known as AKT), both phosphorylate WNK kinases and therefore stimulate SPAK/OSR1 to activate renal ion channels including: NKCC2, NCC, ENaC, and ROMK [11]. These sodium and potassium reabsorption channels in renal tubules are physiological determinants of blood pressure control, and dysregulated WNK pathways central to hypertension-related disorders have recently been under intense investigation [12].

## 2. Familial Hypertension Stemming from Genetic Defects in WNK Signaling Network

### 2.1. WNKs in Pseudohypoaldosteronism Type II (PHAII)

Pseudohypoaldosteronism type II (PHAII), also known as Gordon’s Syndrome or familial hyperkalemic hypertension (FHHt), is an autosomal dominant disorder characterized by salt-sensitive hypertension, hyperkalemia, and hyperchloremic metabolic acidosis. Mutations of *WNK1*, *WNK4*, *KLHL3*, and *CUL3* have been identified to result in the overactivation of NCC and NKCC2, which boosts the sodium reabsorption capacity in the distal convoluted tubule (DCT) and decreases the secretion of K^+^ by ROMK in the following nephron segment (CCD), leading to PHAII [13,14].

From the genetic perspective, PHAII is classified into five genetic subtypes (PHAIIA-E) according to distinct mutation loci [7]. Briefly, PHAIIB and PHAIIC are caused by the mutation of WNK4 (17q21.2) and WNK1 (12p13.33), respectively. PHAIID results from a mutation at 5q31.2, encoding the Kelch-like 3 protein (KLHL3), and an exon 9 deletion of cullin 3 (CUL3) PHAIIE [14,15]. Lastly, the mutation of chromosome 1 (1q31–q42) causes PHAIIA; however, the specific gene that is mutated has not yet been identified [16]. In short, mutations of WNK1, WNK4, KLHL3, and CUL3 impair renal ion channel regulation, thereby initiating PHAII.

**Figure 1:**
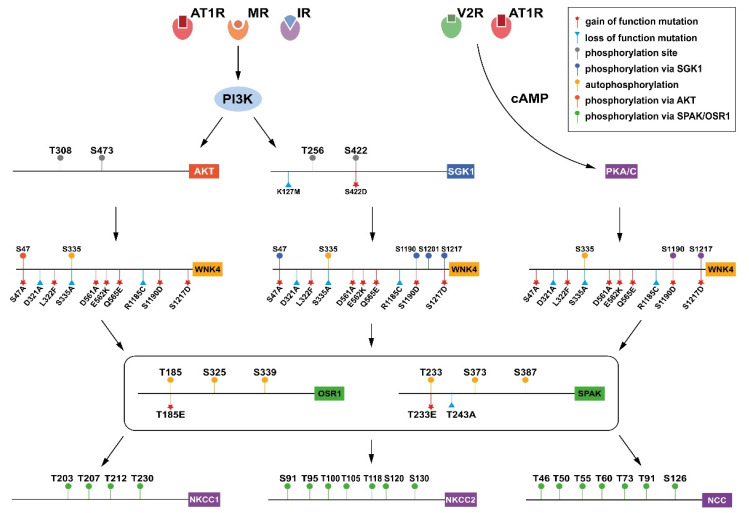
WNK-centric PTM network regulation in renal channels.

**Figure 2:**
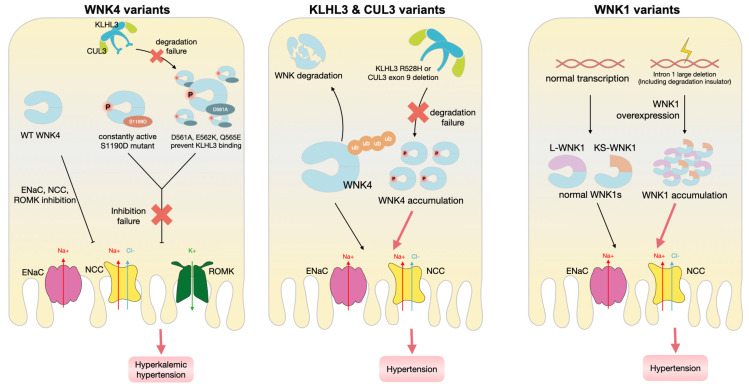
Pathophysiological changes induced by the genetic variants of WNK signaling pathway on the regulation of hypertension and/or electrolyte homeostasis via modulating ion channels on the nephron epithelium. Thick red arrow: pathological result of corresponding mutation; thin black arrow: result of normal physiological condition.

The genes mutated in PHAII, including *WNK1*, *WNK4*, *KLHL3*, and *CUL3*, all encode regulatory proteins implicated in the WNK/SPAK-OSR1/NCC pathway [14]; thus, the mutations in these four genes may lead to the dysregulation of renal reabsorption, causing severe salt-sensitive hypertension [17,18]. Functionally, WNK1 and WNK4 are NCC activators that act via phosphorylating SPAK/OSR1 [11]. Therefore, the overactivation of WNKs may overactivate NCC, thereby increasing ion accumulation, water retention, and hypertension in PHAII. Moreover, as WNKs are targeted by the KLHL3-CUL3 E3 ligase-mediated ubiquitination, insufficient degradation by the KLHL3-CUL3 axis overactivates NCC due to WNK accumulation. The KLHL3-CUL3 E3 ligase-mediated ubiquitination is ablated by mutations, such as KLHL3 R528H, mouse WNK4 acidic motif D561A, Q565E, and E562K (which inhibits KLHL3-WNK4 binding), as well as the deletion of exon 9 in CUL3 (which causes defects in WNK degradation) (Table 1, Figure 1 and Figure 2) [19,20].

The massive accumulation of WNK4 mutants in the PHAII nephrons also affects other electrolyte transport channels, such as ROMK, ENaC, and NCC. In WNK4 PHAII mutant transgenic mice, ROMK and ENaC activity is severely inhibited in the DCT segment 2 (DCT2) and connecting tubule (CNT)—an aldosterone-sensitive nephron segment [78]. ROMK was originally suspected to be inhibited in PHAII due to the low Na^+^ delivery to the lumen because of the NCC overactivation [78]; however, recent studies reported that ROMK is inhibited by PHAII mutant, WNK4, via clathrin-dependent endocytosis, causing hyperkalemia in PHAII, and is not related to WNK4 kinase activity [79].

Thiazide diuretics represent the traditional treatment option for PHAII, which reduces blood pressure by inhibiting NCC-dependent NaCl reabsorption [80]. However, the long-term adaptation to diuretics treatment increases the membrane density of NCC and causes multiple side effects, including hypomagnesemia, hyperuricemia, hyperglycemia, and increased risks of type 2 diabetes [81,82]. Therefore, more small molecule inhibitors are now under intense investigation for potential alternatives for blood pressure control. Several molecules were identified that target the CTD allosteric pocket of SPAK, repressing its binding and thus its activation by upstream WNKs, including STOCK1S-50699, STOCK2S-26016, STOCK1S-14279, Rafoxanide, Clostanel, and the novel hybrid, ZT-1a. Being 79% identical to SPAK, OSR1 is also found to be blocked by STOCK1S-14279 and Closantel [83,84,85,86].

### 2.2. The SGK1-WNK-SPAK/OSR1 Phosphorylation Pathway

**Figure 3:**
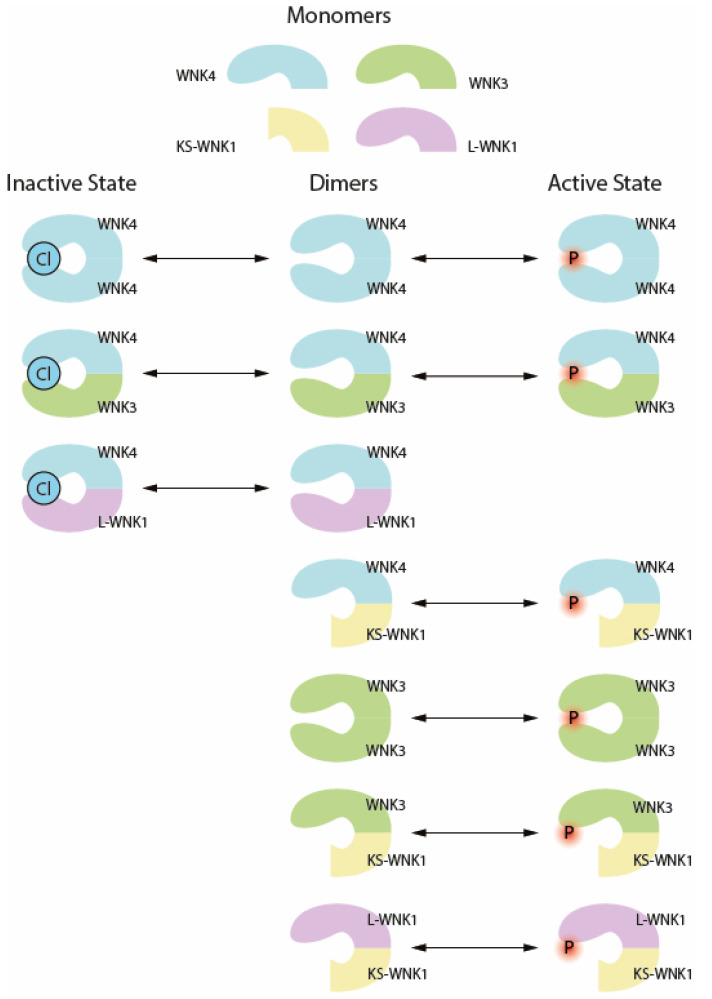
Dynamics of WNK dimer regulation via phosphorylation and chloride binding. Black arrows: state transition catalyzed by the presence of chloride ion.

WNK kinases expressed in the kidney, including WNK1, WNK3, and WNK4, but not WNK2, are responsible for the regulation of multiple ion channels in distal segments of the renal tubules [28]. WNK1–4, containing a conserved serine-threonine kinase domain with no catalytic lysines, possesses an autoinhibitory domain located at the C-terminal region [36]. In addition, a highly-conserved HQ motif of 10 residues at the coiled-coil domain (CCD), demonstrated by Thastrup et al., is essential for the activation-inhibition switch of WNKs through dimer formation. The activation of WNKs is mediated by both dimerization and phosphorylation. Specifically, WNKs’ homo- or heterodimer formation induces the conformational change of WNK kinase, which in turn facilitates autophosphorylation at the T-loop activation kinase domain, leading to WNK activation [28]. Thus, mutations at the T-loop activation kinase domain, such as D321A or D321K-K186D, ablate WNK kinase activity [32]. In addition, mutations of catalytic homologous serine, including S335A of WNK4 and S382A of WNK1, eliminate their phosphorylation activity. The ultimate NKCCs (sodium reabsorption channels) activator, SPAK/OSR1, could also be stimulated by WNK1. WNK1 was documented to have plethoric isoforms as a result of robust alternative splicing [87]. Among the WNK1 isoforms, kidney-specific WNK1 (KS-WNK1) is a member that is expressed only in the renal cortex [88]. As identified in a GWAS study, the third promoter at the first exon generates a variant that is alternatively spliced between exons 4 and 5, replacing the original exon 4 to exon 4a, and disrupting the kinase domain, thus being unable to activate downstream SPAK/OSR1 via kinase-dependent phosphorylation [87,89]. Although KS-WNK1 could not phosphorylate downstream SPAK/OSR1 due to the absence of a kinase domain, KS-WNK1 mitigated the chloride inhibition on WNKs through forming protective dimerizations with other WNK homologs (Figure 3) [27].

Chloride has been identified as a blood pressure enhancer through a clinical trial early in 1929 by showing that sodium chloride showed a higher pressor effect in hypertensive individuals compared to sodium bicarbonate. This phenomenon was later confirmed in three hypertensive rat models: Dahl salt-sensitive (DSS) rat, desoxycorticosterone acetate (DOCA) salt-sensitive rat, and stroke-prone spontaneously hypertensive rat (SHRSP) [90,91]. Recently, a high anion gap with low serum chloride levels have been demonstrated to increase blood pressure in the National Health and Nutrition Examination Survey (NHANES) study [92]. Also, among the hypertensive patients in West Scotland, higher serum chloride levels were shown to be associated with higher serum chloride levels [93].

Hence, it was shown that chloride is also involved in the regulation of WNK activity. The phosphorylation and activation rate of WNKs is reduced by intracellular chloride concentration. J.C. Chen et al. showed that Cl^−^-insensitive-mutant WNK4 knock-in mice displayed hypertension, hyperkalemia, and hyperactive NCC, suggesting that WNK4 may inhibit NCC through a chloride-dependent manner (Figure 3) [94]. In addition, it was reported that chloride ions bound to the chloride-binding pocket located in the kinase domain inhibited the autophosphorylation and activation of WNK [95]. Studies indicated that WNK4 L322F mutation in the chloride pocket desensitizes WNK4′s binding affinity to Cl^−^ ions, therefore resulting in the increased autophosphorylation of WNK4, even in the presence of a higher chloride concentration [32]. WNK1, WNK3, and WNK4 each express distinct chloride-binding affinities, in which chloride binds most efficiently to WNK4 and least efficiently to WNK3 [27,95]. Under high intracellular [Cl^−^] conditions, WNK4 responds swiftly to the chloride change and assembles into inactive dimers with other WNK monomers (Figure 3). On the contrary, WNK3 is insensitive to chloride inhibition, remaining potent to phosphorylate SPAK kinase and promote surface channels [32]. Also, Eduardo RA et al. co-injected KS-WNK1 with WNK4 into Xenopus oocytes, and the resultant WNK4/KS-WNK1 dimer had a lower Cl^−^ affinity, blunting the inhibitory effect of chloride on the activating T-loop autophosphorylation on WNK4 S335 [27].

Interestingly, it has been reported that the autophosphorylation of Drosophila and mammalian WNK3 and WNK4 can be inhibited by high extracellular potassium levels under constant chloride levels. Although direct potassium binding sites on WNKs have not yet been identified, there could be a potential chloride-independent, potassium-dependent WNK activity machinery in the WNK-centric ion regulation pathway [96].

Mechanistically, WNK phosphorylation is regulated by upstream kinases, including SGK1, AKT, and PKA (protein kinase A)/protein kinase C (PKC) [21,26,97]. AKT regulates blood pressure via the PI3K-AKT pathway, which initiates the angiogenesis process and the regulation of NO-dependent epithelial vasorelaxation [98,99]. As downstream factors of angiotensin II or vasopressin signaling, PKA and PKC are both involved in the phosphorylation of ion channels [100]. PKC is associated with cytokine regulation and vascular insulin resistance in diabetes, whereas PKA was shown to increase uromodulin secretion and WNK activity [26,101]. It has been reported that WNK4 in HEK293T cells is commonly phosphorylated by PKC and PKA in four RRXS* motif loci, whereby the asterisk indicates the phosphorylated serine of the WNK4 (mouse: S64, S1169, S1180, S1196) = (human: S64, S1190, S1201, S1217 from PhosphoSitePlus) [26]. In addition, both S47 and T60 were two conservative phosphorylated sites of WNK1 and WNK4, which induced SPAK/OSR1 activation after being phosphorylated. Furthermore, PKA and PKC both phosphorylate KLHL3, which interferes with its binding to WNKs, preventing WNK degradation (Table 1) (Figure 1) [43,54].

Similarly, SGK1 phosphorylates WNK4 at S1169 and S1196, which abrogates its direct inhibitory effect on ENaC or ROMK expression and increases the activation of SPAK/OSR1 [21]. SGK1 is a serine-threonine kinase that acts in the early phase of the aldosterone response [102]. Aldosterone was the first discovered hormone that regulates the expression and activation of sodium transporting channels [103]. PI3K, which is also the downstream effector of insulin, stimulates the activation of PDK1 after the binding of aldosterone to MR. PI3K not only activates PDK1, but also promotes the mTORC2 cascade, which phosphorylates SGK1 at S422 and AKT at S473 [21,104]. Meanwhile, PDK1 could phosphorylate SGK1 at T256 and AKT at T308, both initiating WNK phosphorylation (Table 1) [105,106].

The interaction between SGK1 and WNKs is bi-directional, and evidence suggested that WNK1 and WNK4, but not WNK3, could positively regulate SGK1 [25,107]. Key evidence provided by Charles J. Heise et al. has shown that WNK loss-of-function mutations reduce SGK1 activation. Within the WNK–SGK protein docking interface, the T58A mutation introduced at the N-terminal proline-rich domain (PRD) preceding the kinase domain of WNK1 resulted in stronger SGK1 binding but weaker SGK1 activation, suggesting that WNK1 binding to SGK1 is not sufficient for its activation.

Despite WNKs playing a pivotal role in blood pressure regulation, a limited number of WNK inhibitors were designed to address the anti-HTN therapeutic effect targeting the WNK family. As for the current date, WNK463 is the only WNK kinase inhibitor showing both in vitro and in vivo therapeutic effects [108]. WNK463 is the first orally bioavailable WNK kinase inhibitor targeting all four WNK family members and the WNK1-catalyzed phosphorylation of OSR1 [108]. Due to its acting as a low-nanomolar competitor to the ATP-binding site in both WNK1 and WNK4, the oral administration of WNK463 significantly reverses blood pressure elevation in hypertensive rats due to overexpression of human WNK1 [108]. However, further investigation is required to elicit its pharmacological profile before commencement of clinical trials.

### 2.3. The WNK Degradation Pathway: KLHL3 and CUL3

Kelch-like 3 (KLHL3) and Cullin-Ring ubiquitin 3 (CUL3) are inhibitors of WNK kinases. KLHL3 is expressed in the apical membrane of the DCT, and CUL3 is expressed along the nephron. KLHL3 is composed of three domains, kelch-like repeats in a beta-propeller structure at the C-terminal, N-terminal BTB and BACK domain—whichfunctions as an adaptor to capture substrates—and CUL3 transfers ubiquitin from the E2 ubiquitin-conjugating enzyme to these substrates, initiating the proteasomal degradation of the substrate [7]. L-WNK1 or WNK4 is captured by the C-terminus of KLHL3 at the kelch-like repeats, and RING-box protein 1 of CUL3 binds to the KLHL3 BTB domain, recruiting an E2 ubiquitin-conjugating enzyme that ubiquitinates L-WNK1 or WNK4 [53]. This causes the two WNKs to be degraded by proteasomes. KLHL3 and CUL3 mutations are the primary causes of PHAII, as 80% of PHAII patients have KLHL3 or CUL3 mutations. PHAII mutations of KLHL3 are found in the kelch repeats, the BTB, and the BACK domain. Mutations in the BTB and BACK domains impair the binding of CUL3, and the mutations in kelch repeats inhibit the capture of WNKs. As demonstrated in KLHL3 R528H/+ mice, the R528H mutation in the kelch repeat region is autosomal dominant, exhibiting overexpressed WNK1 and WNK4 due to the defective degradation complex [12]. However, the KLHL3+/− mice do not express the PHAII phenotype. Moreover, affected by insulin and AngII, the KLHL3 is targeted by the PKC on S433 in the kelch repeats, a site which is also targeted by PKA and Akt. The phosphorylation by PKA/PKC of KLHL4 S433 inhibits its degradation towards WNK4. S433E mutation of KLHL3 is also PHAII-causing, which may also cause the defective binding of WNKs (Table 1) (Figure 2) [19,109].

PHAII caused by CUL3 is due to the production of CUL3 that has an exon 9 deletion (CUL3Δex9), lacking 57 amino acids. The identified PHAII-causing CUL3 mutations cause the defective degradation of WNK kinases, however, it is thought to be a gain-of-function mutation since it is an autosomal dominant mutant that carries dominant negative effects seen in heterozygotes. There are two mechanisms suggesting how Δex9 mutants cause PHAII: First, CUL3Δex9 leads to the degradation of KLHL3, abrogating WNK4 inhibition due to the reduced formation of E3-ubiquitin ligase. This stems from the off-target ubiquitination effect of CUL3Δex9 that is overactivated by NEDD8 neddylation due to the presence of mutations (Figure 2). This is supported by the study done by Yoshida et al. in CUL3Δex9/+ mice, where KLHL3 protein expression is indeed decreased in the kidneys from CUL3Δex9/+ mice. Second, however, as shown in CUL3Δex9 mice, there is decreased CUL3Δex9 expression but normal KLHL3 expression, suggesting that the CUL3Δex9 mutation might also allow the auto-ubiquitination of CUL3Δex9.

## 3. WNK Interactive Channels in the Proximal Tubule (PT)

### 3.1. Na^+^-H^+^ Exchanger 3 (NHE3)

NHE3 is an antiporter that is mostly expressed at the apical side of the proximal tubule (PT) that carries one sodium ion (Na^+^) into PT and brings out one hydrogen ion (H^+^) to the urine filtrate. NHE3 plays a significant part in upregulating sodium transport in the brush border of the intestinal system, reproductive system, and especially in the proximal tubule, and it also mediates blood pH values [110,111].

NHE3 at the apical renal tubule is upregulated by the renin-angiotensin-aldosterone (RAAS) system, the canonical hypertension-driving mechanism [112]. Xiao C. Li et al. showed that increased NHE3 was associated with hypertension and revealed that the NHE3 inhibitor reduced angiotensin II (Ang II)-induced hypertension in PT-Nhe3−/− mice, implying the regulatory effect of Ang II on NHE3 [113]. In fact, recent studies have shown that not only can Ang II enhance NHE3 transcription, but it can also directly activate NHE3 functioning through Protein Kinase C-alpha (PKC-alpha) [114]. Thus, orally-administered small molecule NHE3 inhibitors, such as AVE0657 [113] and AZD1722 (Tenapanor), have shown potent effects on the blockage of either renal or intestinal afferent Na^+^ absorption and control of hypertension [115].

NHE3 is phosphorylated by SGK1, whose transcription is stimulated by glucocorticoids, aldosterone, and insulin through the binding of glucocorticoid receptors (GR), mineralocorticoid receptors (MR), and insulin receptors (IR), respectively [56,116,117,118,119]. This phosphorylation-induced stimulation is sustained by glucocorticoids or PI3K, and thus, SGK1 would stimulate NHE3 as long as it is phosphorylated by glucocorticoid or PI3K [56,117,118,120]. Moreover, insulin can also activate NHE3 either by the PI3K/SGK1 pathway or the PI3K/AKT pathway (Figure 3) [121].

In addition to Ang II and aldosterone in the RAAS system, endothelin-1 (EDN1) and glucocorticoid were also found to trigger NHE3 exocytosis through a RAAS-independent manner [122]. This may partially explain why the enhancement of ET-1 expression is observed in human stage 2 (severe) hypertension, and endothelial injury may lead to vasogenic HTN [123]. Moreover, an acute increase in endothelin-1 may increase NHE3 expression, while the long-term presence of endothelin-1 may chronically decrease the expression of NHE3 [123].

The phosphorylation of NHE3 at mouse S552 and S605 (equivalent to human NHE3 S555 and S607) is suspected to be related to its apical trafficking [124]. In response to vasopressin, the increased cAMP concentration can lead to higher phosphorylation rates of NHE3 S552 via PKA, which has been proved to be inhibited by cyclic GMP kinase II (cGKII) (Table 1) [125,126].

### 3.2. Na^+^/3HCO^3−^ Co-Transporter (NBC1)

NBC1 is an electric potential-driven transporter that is expressed at the basolateral side of the proximal tubule (PT) each time that NBC1 imports one Na^+^ along with 3HCO^3−^ from PT epithelium to the bloodstream. Through the joint effort of NHE3 and NBC1, PT reabsorbs 80–90% of bicarbonate and 65% of sodium ions from the Bowman capsule filtrates. The sodium re-uptake current generated by NHE3 and NBC1 not only regulates electrolyte homeostasis, but also maintains the acid–base balance by HCO^3−^ retrieval. In particular, a recent study showed some congenital type II renal tubular acidosis (RTA) was attributed to NBC1W516X, in which the nonsense-mutated NBC1 transcript underwent nonsense-mediated decay (NMD) [127]. Thus, the absence of NBC1 leads to the failure of reabsorption of sodium and bicarbonate, thereby resulting in proximal tubule acidosis.

Given that NBC1 is a significant regulator of renal homeostasis, NBC1 is regulated by many pathways, for instance, angiotensin II (AngII) extracellular signal-regulated kinases (ERK) activation, insulin signaling, and adrenergic stimuli [128]. One study showed that insulin stimulated the expression of NBC1, thus resulting in hypertension. This could reveal why hyperinsulinemia facilitated by insulin resistance may cause sodium retention and finally hypertension (Figure 3) [129]. In addition, adrenergic hormones, such as glucocorticoid and noradrenaline, may stimulate the expression of NBC1 and thus cause hypertension; however, the chronic effects of such adrenergic activation on blood pressure are still unspecified [130].

Multiple regulating pathways have suggested novel therapeutic targets in human hypertension, as reviewed by Motonobu Nakamura in 2014 [131]. However, the channel is still not fully researched and there are no inhibitors, drugs, or therapy targeting NBC1.

## 4. WNK Interactive Channels in the Thick Ascending Limb (TAL)

### 4.1. Na/K/2Cl Co-Transporter 2 (NKCC2)

The TAL is responsible for about 25% of the renal sodium reabsorption, which is mostly carried out by the type 2 Na^+^/K^+^/2Cl^−^ co-transporter (NKCC2 or BSC1) [132]. NKCC2 assists transporting sodium from filtrate into the epithelial cells, bringing in one sodium ion, one potassium ion, and two chloride ions, thereby mediating approximately 90–100% of Cl^−^ reabsorption and ~50% of Na^+^ transport in the TAL [133]. NKCC2 is encoded by the *SLC12A1* gene, which undergoes splicing, translation, sorting, and trafficking to the apical side of the TAL by intracellular vesicles. Three common SLC12A1 exon 4 alternative spliced variants, NKCC2A, NKCC2B, and NKCC2F isoforms, differ in their distribution along the nephron and their affinity to Cl^−^. NKCC2B, located mainly in the cortical TAL, has the highest affinity for Cl^−^, and NKCC2F, located in the medullary TAL, has the lowest [134,135]. NKCC2A loss in mice exhibited low Cl^−^ reabsorption and a compensatory high NKCC2B expression, as shown by Mona Oppermann et al. [136]. Recent breakthroughs were made on the molecular pathways underlying NKCC2, including its apical membrane trafficking, interaction partners, phosphorylation cascade, and its interconnection with the regulatory hormones of ion homeostasis.

NKCC2 was linked to blood pressure regulation early in the Framingham Heart study (FHS). A re-screening of *SLC12A3* (NCC), *SLC12A1* (NKCC2), and *KCNJ1* (ROMK) identified several SNPs that cause blood pressure dysregulation [137]. For instance, a disruptive mutation in R302- was shown to be disease-causing and has an allele frequency of 0.0069 in European Caucasian disease patients. Additionally, Y1070C, R302W, and L505V mutations all resulted in low NKCC2 activity. Furthermore, mutations of NKCC2 and the malfunction of its regulators are known to cause Bartter Syndrome, a salt-wasting hypotensive condition with reduced UMOD levels [138,139]. However, recent evidence indicated that genetic alterations in the upstream regulator of NKCC2 are related to salt-sensitive hypertension in both animal models and clinical settings [140,141,142]. Moreover, the degradation of NKCC2 interfered with melanoma-associated antigen D2 (MAGE-D2) and is stimulated by the osteosarcoma amplified 9 proteins (OS9). The MAGE-D2 loss of function mutation was shown to cause Bartter’s Syndrome due to the decreased interference of NKCC2 degradation [143]. On top of Bartter’s Syndrome, NKCC2 is also the terminal effector of PHAII, an autosomal dominant inherited disease that displays hypertension and hyperkalemia (Table 1) (Figure 3) [14].

As shown in previous studies, the membrane expression of NKCC2 is mediated by a dynamic network of continuative exocytosis that occurs along with endocytosis, suggesting that the regulation of NKCC2 largely depends on subapical vesicle transportation [144]. The sorting and trafficking of NKCC2 to the apical membrane has been proved to be mediated by PTMs, such as phosphorylation. WNK-SPAK/OSR1 pathway-directed phosphorylation is the most important NKCC2 activator. WNK1, WNK3, and WNK4 can phosphorylate SPAK and OSR1, which further phosphorylate the N-terminal regulatory regions of NKCCs (including NKCC1, NKCC2, and NCC) [145]. OSR1 is the main driver of NKCC2 phosphorylation, and SPAK is mainly responsible for NCC phosphorylation [146,147,148].

S91, T95, T100, T105, S118, S120, and S130 are conserved sites that are phosphorylated by SPAK/OSR1 at the cytoplasmic NH2 tail via interaction with the RFQV domain, thereby enhancing NKCC2 vesicle trafficking to the cell surface and accelerating the intake of sodium ions that cause a blood pressure raising effect [62,149,150,151,152,153,154].

Phosphorylation-directed NKCC2 transportation is also largely regulated by AVP, which is the most studied NaCl transport activator in TAL [155]. In response to the binding of AVP to V2R, cyclic adenosine monophosphate (cAMP) is activated, stimulating the PKA-mediated NKCC2 phosphorylation at N-terminal S87 (equivalent to hNKCC2 S91), T96 (equivalent to hNKCC2 T100), S126 (equivalent to hNKCC2 S130), and C-terminal S874 (equivalent to hNKCC2 S879), which upregulates the activity of NKCC2 in rat cortical membrane cells and renal medullary thick ascending limb cells (mTAL) [61,125,156].

**Figure 4:**
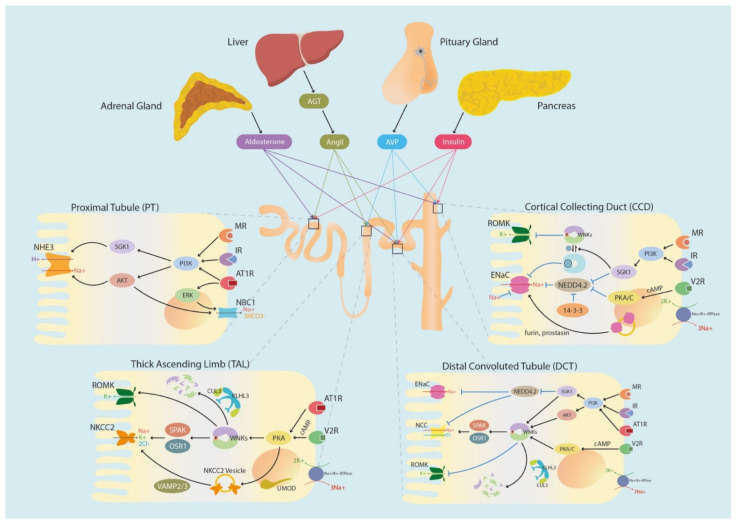
Hormonal and cellular regulation pathway of ion channels along renal segments.

After the conduction of AVP-V2R-cAMP-PKA pathway phosphorylation, NKCC2 in vesicles are delivered to the apical membrane by *N*-ethyl-maleimide-sensitive factor attachment protein receptor (SNARE) family members, such as vesicle-associated membrane protein 2/3 (VAMP2/3) [157]. In summary, PKA, the downstream of AVP stimulation, enhances NKCC2 vesicle shuttling by phosphorylation-induced vesicle-trafficking proteins binding and the SNARE-associated fusion of vesicles to the apical membrane (Figure 4) [151,158].

Previously, AMPK signaling was also considered a pathway that phosphorylates NKCC2, but at much lower levels. In vitro studies carried out by Richardson et al. in HEK293 cells exhibited increased NKCC2 S130 phosphorylation when treated with phenformin, the AMPK activator, whose effect was eliminated by a non-selective AMPK inhibitor. In addition, S91, T95, T100, T105, and S130 were also identified as being phosphorylated (Figure 1) (Table 1) [154,158]. Interestingly, though stimulating sodium reabsorption in the TAL, AMPK signaling in the CCDs decreases cAMP-dependent ENaC activation via the ubiquitin ligase NEDD4.2 [159,160].

Sorting-related receptor with A-type repeats (SORLA), which is an intracellular receptor involved in diverse protein sorting and trafficking, is also identified to regulate NKCC2 phosphorylation [161]. The generation of SORLA KO mice provided clues for linking SORLA and NKCC2 function. SORLA KO mice displayed decreased NKCC2 phosphorylation at the SPAK/OSR1-dependent T96 and T101 due to the interference of SORLA with the function of NKCC2 dephosphorylation agent, calcineurin Aβ (CnAβ). The phenomenon could be rescued by cyclosporine, the calcineurin inhibitor [162].

Other PTMs are also known to be related to NKCC2 trafficking. The N-linked glycosylation of N442 and N452 located between transmembrane domains 7 and 8 mediated trafficking activation. The ablation of glycosylation on these two sites in Xenopus laevis oocytes showed a decrease in NKCC2 plasma membrane abundance, while it did not alter total cellular NKCC2 levels [158,163]. Despite the discovery, the mechanism of how glycosylation mediates NKCC2 trafficking remains unknown. In addition, the question of why a fraction of non-glycosylated NKCC2 still moves to the cell surface requires further investigation.

Several previous studies have indicated that NKCC2 phosphorylation is more significant under low extracellular chloride concentrations and hypotonicity due to unknown causes. However, recent studies have proposed the mechanism of intracellular chloride inhibiting WNK kinases, raising the importance of intracellular electrolyte homeostasis in blood pressure control [64,94,152,154,164].

The ion exchange in NKCC2 is affected by the transmembrane gradient of Na^+^, K^+^, and Cl^−^; in a sense, the NKCC2 activity was regulated by K^+^ homeostatic protein Na^+^-K^+^/ATPase, epithelial K^+^ channels (ROMK), and chloride exiting channels (CLCNKB) [165]. After sodium ions are transported into TAL cells by the NKCC2 from the apical membrane, the primary exit pathway would be the Na^+^-K^+^/ATPase at the basolateral membrane, supporting the driving force of NKCC2 transportation. For example, a high-salt diet could suppress Na^+^-K^+^/ATPase activity in the kidney, causing the intracellular accumulation of sodium ions, thus inhibiting NKCC2 activity and finally causing hyperchloremia [166,167,168]. However, in vivo studies in Dahl salt-sensitive rats, which were fed with a high-salt diet, have an increased NKCC2 surface abundance and sodium reabsorption rate [169]. ROMK generates a potassium ion gradient to facilitate NKCC2 membrane targeting, thereby putting ROMK in the regulation of NKCC2 surface targeting and cooperates with NKCC2 to transport electrolytes via excreting K^+^ to the urine, creating a K^+^ concentration gap across the apical membrane, and further facilitating NKCC2 transportation. This indicates the modulating role of ROMK activity in sodium reabsorption [170].

NKCC2 is also regulated by cytokines like tumor necrosis factor-α (TNF-α). Under low chloride hypotonic stress, UMOD, a filamentous GPI protein secreted in urine, was demonstrated to increase the phosphorylation rate of NKCC2 in UMOD-/- mice and rabbit TAL cells [139]. As shown in previous studies, TNF-deficient mice (TNF^−/−^) displayed increased total NKCC2, and it was also further identified that NKCC2A isoform mRNA was downregulated by TNF-α [171]. This could be linked to the NKCC2 upregulating effect via UMOD, a filamentous GPI protein secreted in urine. Under low chloride hypotonic stress, UMOD was demonstrated to increase the phosphorylation rate of NKCC2 in THP-/- mice and rabbit TAL cells, as its high affinity to TNF-α secreted in the TAL prevents NKCC2 inhibition [139].

### 4.2. Renal Outer Medullary Potassium (ROMK) Channel

The renal outer medullary potassium channel (ROMK), also known as Kir1.1, is a voltage-dependent K^+^ channel that is expressed on the apical side of the renal tubule, such as in TAL, DCT, and CCD [172]. The activity of ROMK is largely dependent on a K^+^ gradient or membrane potential tuned by other ion channels. While being mostly expressed in TAL, ROMK is involved in blood pressure regulation by balancing the K^+^ gradient when NKCC2 reabsorbs Na^+^, hence ROMK gain-of-function mutations may indirectly promote hypertension through NKCC2 function [70,173,174]. Meanwhile, ROMK gain-of-function mutations also generated increased K^+^ efflux, largely decreasing the intracellular K^+^ concentration. Cation channels in nephron epithelial cells, including NHE3, NKCC2, NCC, and ENaC, increase their absorption to compensate for the negative membrane potential resulting from ROMK gain-of-function. The increase in sodium and hydrogen intake results in hypertension and metabolic acidosis [175,176].

Genomic sequencing from the Framingham Heart Study Offspring cohort surprisingly revealed that patients with ROMK loss-of-function polymorphisms (R193P, H251Y, T313FS, P166S, and R169H) are less susceptible to hypertension. Following the sequencing results from the Framingham study, Liang et al. examined those loss-of-function ROMK mutations in Xenopus oocytes by applying two microelectrode voltage clamps. However, the results of the in vitro electrophysiology experiments linked only 3 out of 5 ROMK SNPs, which are R193P, H251Y, and T313FS, to reduced ROMK channel activity. It is worth noting that ROMK T313FS is frequently found in salt-wasting and hypotensive Bartter’s Syndrome [177]. Moreover, the T313FS mutation was later found toaffect post-translational glycosylation in the C-terminus of ROMK, with R193P and H251Y mutations also displaying identical immature glycosylation as T313FS does. Three of the ROMK mutants are all less surface-targeted, indicating that the post-translational C-terminus glycosylation of ROMK may play a role in its membrane trafficking [170]. 

Nevertheless, the other two hypertension-resistant mutations, the ROMK P166S and R169H mutants, did not manifest a similar glycosylation defect [170]. However, in an independent article, P166S and R169H were reported to be at the adnexa of the phosphatidylinositol 4,5-bisphosphate (PIP2)-binding domain. PIP2 binding alongside PKA phosphorylation, ATP binding, and intracellular pH (pHi) are essential prerequisite factors for maintaining maximal ROMK activity. Hence, the P166S and R169H mutant ROMK with defective PIP2 binding may lead to an impaired inward-rectifying potassium current ROMK [178]. Collectively, distinct molecular genotypes with common physiology phenotypes may arise from drastically different molecular mechanisms. In the case of ROMK mutations in the Framingham study, ROMK R193P, H251Y, and T313FS caused hypertension resistance by ablating C-terminal glycosylation to ablate molecule trafficking, whereas P166S and R169H gain hypertension resistance through the abrogated PIP2 in the context of channel gating.

ROMK is a three-member splice variant family, where splice variants differ in their regulation due to the variation of mRNA 5′-coding and 3′-non-coding regions [10]. The membrane abundance of ROMK is regulated by extracellular stimuli, such as aldosterone and AVP, where differences in the regulation pathways exist. Studies have shown that the membrane abundance of different ROMK variants is activated by SGK1 and PKA in several ways. First, ROMK1 is inhibited by PKC via direct S4 and S17 phosphorylation, while ROMK2 does not possess such sites due to a truncated N-terminus [10]. Though ROMK3 interacts with PKC by the extended N-terminus that contains a PKC-targeting threonine residue, it is not homologous to the site of ROMK1 (Table 1). This supports the fact that ROMK1 is inhibited by PKC, while ROMK2/3 inhibition is not due to structural differences. 

Secondly, there were studies reporting that ROMK1 is phosphorylated and activated by SGK1 and PKA at S44, whose activity is induced by aldosterone and AVP, respectively. Further studies applying phosphorylation site mutations also indicated that phosphorylation null mutation S44A and phosphorylation-mimicking mutation S44D had opposite effects on the cell surface density of ROMK. This supports the notion that S44 is an important channel activation site that is correlated with plasma membrane abundance, an effect mediated by the trafficking/transport protein Na^+^/H^+^ exchange regulatory factor 2 (NHERF2). Moreover, ROMK1 S44 phosphorylation increases electrophysiological function at cytosolic pH 6.6–7.3, which is more acidic than at normal conditions. Despite activating ROMK1 directly, S422-activated SGK1 also phosphorylated WNK4 at S1169/S1196 (hWNK4 = S1190/S1217), which alleviated the ROMK2 endocytosis by WNK4 (via the C-terminal NPXY-like motif). The S1169D phosphorylation-mimicking mutant does not inhibit ROMK surface expression, just like the effect of wild-type WNK4 when it is phosphorylated by SGK1. In contrast, the WNK4 PHAII mutant, which cannot be degraded by the KLHL3/CUL3-RING ubiquitin complex, manifests enhanced ROMK2 endocytosis (Table 1) (Figure 2).

Meanwhile, p-S422 SGK1 and p-473/p-308 AKT somehow induce an intersectin/dynamin-dependent ROMK1 endocytosis rate via phosphorylating WNK1 at T58 [21,107]. The SGK1 constitutive phosphorylation mutant, S422D, largely increases the phosphorylation rate of WNK1 T58, which in turn reduces ROMK1 cell surface abundance and is alleviated by dominant-negative intersectin/dynamin co-expression. Moreover, Chih-Jen Cheng et al. also demonstrated that both the wild-type ROMK1 and phosphorylation-mimicking S44D ROMK1 mutant are decreased by the S422D SGK1 mutant. However, it is still unclear how and why SGK1 exerts contrary effects on ROMK1 [34]. Similar to WNK1 and WNK4, WNK3 is also involved in ROMK1 endocytosis via its C-terminus in a kinase-independent manner. What is different is that WNK3 does not affect ENaC activity in the Xenopus laevis oocytes injection experiment [179]. In addition, as the molecular mechanisms of WNK3 have not been fully investigated, there is still no report regarding the upstream factors of WNK3 that induce ROMK1 endocytosis (Figure 1 and Figure 4).

On top of WNK-dependent endocytosis, the ROMK surface expression was shown to be associated with a K^+^ diet. Lei Yang et al. showed in their study that ROMK was constitutively active and secreted the most potassium in the CNT, and ROMK activity in the CNT was increased with the high-K^+^ diet and decreased with a low K^+^ food intake. In this regard, ENaC also positively responds to the increased K^+^ current. Meanwhile, ROMK and ENaC activity increase in the downstream CCD. These data support the idea that a high-K^+^ diet could increase the risk of hypertension due to overactivated sodium reabsorption channels [180]. Another study conducted in rats reported that heterozygous ROMK disruption may lead to reduced blood pressure and less severe renal injury. A human genetic study also discovered that ROMK deficiency brings about hypertension protective characteristics, which is consistent with the Framingham Heart Study [181]. Furthermore, homozygous loss-of-function mutations of ROMK are associated with type II Bartter’s Syndrome, thereby causing polyuria, renal salt wasting, and hypotension [182]. These clinical implications emphasize the important role of ROMK in hypertension.

Although pathologies of ROMK dysregulation have been widely studied, more detailed ROMK regulation mechanisms remain unclear. ROMK family members are distributed unevenly in different segments of renal tubules: the DCT expresses ROMK2/3, the CNT expresses ROMK2, and the CCD expresses ROMK1/2 [10]. It is possible that different nephron segments contribute to the different effects of potassium transportation due to the diverse effects of various pathways that target distinct ROMK splicing variants. ROMKs are multifactor-regulated ion channels, their activity is affected by cellular kinases and extrinsic and intrinsic factors that may regulate ROMK at multiple statuses.

Collectively, ROMK promotes hypertension through reabsorbing sodium ions with NKCC2 and ENaC, hence, targeting ROMK could plausibly ameliorate high blood pressure. An acute pharmacological intervention introduced novel ROMK inhibitors, compound A, MK-7145, and MK-8153 [182,183], thereby reducing ROMK activity and therefore ablating the function of coupled sodium reabsorption by NKCC2 and ENaC, which has been proved to be a novel therapeutic pathway for hypertension.

### 4.3. Uromodulin (UMOD)

Uromodulin (UMOD), otherwise known as Tamm–Horsfall glycoprotein (THP), is a glycophosphatidylinositol (GPI)-anchored protein that is mostly expressed at the apical side of the TAL and minimally expressed at the DCT, making it the most abundant protein in urine under normal physiological conditions [184,185,186,187]. Functionally, uromodulin forms a high-molecular-weight filamentous skeleton via the formation of aggregated polymers, which is regulated by three epidermal growth factor (EGF) domains and one zona pellucida-like domain [188]. The equipped GPI-anchor of uromodulin at the C-terminus performs its function through assisting vesicle trafficking to the lipid rafts, which facilitates the trafficking of NKCC2, and possibly ROMK and NCC, from the basal to the apical side of epithelial cells via shared lipid rafts [139,189,190,191,192].

As uromodulin is excreted in bulk to urine by the TAL, it is not surprising that it plays an essential role in the regulation of TAL-localized ion channels, such as NHE3, Na^+^-K^+^-ATPase, NKCC2, ROMK, NCC, and ENaC [185,186,193,194]. It has been shown to escort SPAK/OSR1 to phosphorylate NKCC2 in the apical membrane, preferably at Ser91, Thr95, Thr100, Thr105, and Ser130 (Figure 4) [154]. In hypertension-mimicking Dahl salt-sensitive rats, aberrant salt reabsorption was found with increased NKCC2 expression as well as NKCC2 phosphorylation (5-fold compared to the Dahl salt-resistant control) [140]. In parallel, Kerim Mutig et al. demonstrated that UMOD-/- mice displayed salt-wasting Bartter’s Syndrome-like phenotypes and had lower phosphorylation levels of NKCC2 [139]. In contrast, mice with overexpressed uromodulin showed salt-sensitive hypertension. In conclusion, uromodulin-facilitated NKCC2 phosphorylation contributes to the hypertension phenotype, especially under low [Cl^−^] levels.

In addition, the NKCC2 phosphorylation and activity was also regulated by the dDAVP-V2R-cAMP-PKA pathway; namely the dDAVP promotes salt reabsorption through the upregulation of p-NKCC2 [149]. However, the abrogated NKCC2 phosphorylation in UMOD-/- mice conferred a dDAVP-resistant phenotype in salt reabsorption, indicating that the uromodulin-modulated SPAK/OSR1 kinase activation on NKCC2 superimposed dDAVP ligand activation on salt regulation.

Apart from NKCC2 regulation, there are studies suggesting that uromodulin regulates NCC phosphorylation in HEK293 cells possibly by modulating the SPAK/OSR1 signaling pathway. THP also increased the sensitivity of NKCC2 to intracellular chloride depletion in the presence of WNK3, a strong activator of NKCC2, which can also activate NCC [139].

UMOD-/- mice also displayed a significant decrease in ROMK plasma membrane abundance, and co-expression of ROMK with uromodulin enhanced ROMK-mediated K^+^ transport [195]. Further identification of the mechanism underlying the interaction between ROMK and uromodulin showed that uromodulin ablates the C-terminal ubiquitin-binding targeting ROMK, and also cooperates with ROMK in subapical vesicle shuttling [196]. ROMK is also involved in the salt-wasting condition of Bartter’s Syndrome, as ROMK works cooperatively with NKCC2, the sodium reabsorbing channel. The disruption of the ROMK-regulated K^+^ current caused by defective uromodulin function would severely abrogate NKCC2 activity [166].

The image of uromodulin being implicated in electrolyte channel transporting had brought forth the role of uromodulin in hypertension. Thus, the mutations of UMOD have recently been linked to the pathophysiology of blood pressure control. Previously, various GWAS and genetic studies further investigated the BP phenotypes of families possessing certain variants of UMOD. SNPs located in the promoter region of UMOD genes, such as rs12917707, rs1333326, and rs4293393, are associated with hypertension risks and chronic kidney diseases (CKD), which is indicative of being related to higher renal uromodulin expression, urinary secretion, altered eGFR, and impaired renal function [156,157,158,159,160,161].

rs4293393, a common UMOD C allele SNP, has a frequency of prevalence ranging from 70–80% in Africans and Europeans and can be >90% in East Asians [197,198]. It featured significantly lower uromodulin expression levels, which caused higher eGFRs [199] and in turn lowered the risk of CKD, the formation of kidney stones, and hypertension [197]. Furthermore, the T allele of rs12917707 [193], which is located 3.4 kb upstream of UMOD, is shown to have increased urinary uromodulin levels. It has also been shown to lower the risk of CKD-related hypertension by about 20%, as it was shown to elevate both the glomerular filtration rate reckoned by serum creatinine (eGFRcrea) and cystatin C (eGFRcys) [200].

Furthermore, the minor G allele of the variant near UMOD rs13333226 is shown to protect individuals against monogenic hypertension in European communities, however, in a similar study conducted in black South African populations, SNP rs1333326 was shown to have no significant differences in genotype frequencies between chronic kidney disease (CKD) cases and control groups, implying the genetic differences in different ethnic groups [201].

A pathogenic mutant of UMOD would possibly cause the dysregulation of glomerular renal filtration, resulting in severe hypertension. Disease-causing autosomal dominant rare UMOD mutations C315F, W202S, and C106F are reported in families with focal segmental glomerulosclerosis (FSGS) or hereditary glomerular disease, thereby linking uromodulin function to broader renal function regulation [196,202,203]. However, the mechanism of such mutations is still unclear.

## 5. WNK Interactive Channels in the Distal Convoluted Tubule (DCT)

### Na^+^-Cl^−^ Co-Transporter (NCC)

The Na^+^-Cl^−^ co-transporter (NCC), which is encoded by the *SLC12A3* gene, is the most representative thiazide-sensitive Na^+^-Cl− co-transporter that is expressed on the apical side of DCT [12,21,204,205,206]. NCC contributes to renal electrolyte transport by reabsorbing one sodium ion along with a chloride ion, and is ablated by thiazide diuretics. However, the specific amino acid residues that thiazide binds to inhibit NCC remain unknown [207,208].

Pathologically, the loss-of-function mutations N59I, R83W, I360T, C421Y, G463R, G731R, L859P, and R861C of NCC were identified in the patients of Gitelman Syndrome, whereas overactivation of NCC was shown to lead to hypertensive PHAII [209,210]. Therefore, NCC regulation is important in blood pressure control. Mechanistically, the activity of NCC is controlled by multiple cellular pathways, such as the WNK signaling pathway. After vasopressin, such as arginine vasopressin (AVP), binds to the V2 receptor (V2R), PKC or PKA are activated, and this results in the phosphorylation of WNK, which triggers the activation of SPAK or OSR1, and, lastly, the phosphorylation of Thr55 and Thr60 at the N-terminal of NCC, thereby enhancing sodium reabsorption [26,211,212]. Therefore, the phosphorylation of Thr60 of NCC would cause an increase in volume and later increase blood pressure, whereas the loss-of-function mutation, T60A, may lead to salt-wasting Gitelman Syndrome in Han Chinese people due to its defective phosphorylation and enhanced degradation of NCC [213]. On the contrary, another study has shown that the T60D gain-of-function mutation that mimics phosphorylation increases the membrane stability of NCC in MDCK cells, therefore reducing the degradation rate of NCC after cycloheximide treatment (Figure 1 and Figure 4) [214].

The stimulation of NCC is initiated by Ang II, aldosterone, insulin, and vasopressin. After the binding of arginine vasopressin (AVP) to the V2 receptor (V2R), PKC or PKA are activated and phosphorylate WNKs, which enables the further phosphorylation of NCC by the SPAK/OSR1 axis. The activated SPAK and OSR1 activate NCC by phosphorylating Thr46, 55, 60 (human) [11,211]; Ser73, 91, 126 (human) [11,145,215]; Thr48, 53, 58 (mouse and rat) [11]; and Ser71, 89, 124 (mouse and rat) [216], thereby showing that WNK has a stimulating effect on NCC. Interestingly, Thr60 was shown by several studies to be a critical regulator of NCC activity, as changing T60 to a non-phosphorylated amino acid (T60A) prevented or reduced phosphorylation at other sites (T48, T55, S73, or S91) (Table 1) [11,205,214,217].

## 6. WNK Interacting Channels in the Cortical Collecting Duct (CCD)

### 6.1. SGK1-NEDD4L Degradation Mechanism

The epithelial sodium channel (ENaC) is responsible for sodium reabsorption in distal nephrons. Overactivation of ENaC can cause hypertension—in the case of the autosomal dominant Liddle’s Syndrome [218]. The binding of the ENaC proline-rich PY motif to the WW domain in the NEDD4.2 E3-ligase initiates ENaC ubiquitination at the intracellular C-termini within the α, β, and γ subunits, and the surface ENaC degradation in turn reduces epithelial Na^+^ intake and later water retention, which results in hypertension [71,72,219,220,221,222]. The degradation of ubiquitinated surface ion channels was also reported in the axis of NEDD4.2-mediated ubiquitination of NCC and NKCC2 in the kidney, as well as NKCC1 in the colon [223,224]. Interestingly, the NEDD4.2-dependent ENaC degradation is abrogated when NEDD4.2 is phosphorylated at S342 by SGK1 and other unspecified phosphorylated T367 and S448 sites within WW domains (Table 1) [47]. It was suggested that upon phosphorylation, NEDD4.2 increases its binding affinity to 14-3-3 protein, which perturbs its binding capacity to ENaC, hence preserving ENaC surface expression [46,48]. In other words, SGK1-propagated PTM (phosphorylation on NEDD4.2 and ubiquitination on ENaC) cascade increases hypertensive sodium uptake by blocking NEDD4.2-mediated ENaC resorption. In addition to SGK1, NEDD4.2 was also found to be phosphorylated at S327, S221, and T246 by other kinases (Table 1) [50]. PKA phosphorylates NEDD4.2 and blocks its inhibitory effect on ENaC through the cAMP–PKA pathway. Similar to the role of SGK1 and PKA kinases, deubiquitinating enzymes (DUBs), such as UCH-L3, were also found to counteract NEDD4.2 by deubiquitinating ENaC, thereby promoting the surface targeting and sodium reabsorption of ENaC. Since the ubiquitination of the surface ENaC channel is reversible, it indicates that the ion current is controlled by a complex network with pertinent signal coordination (Figure 4) [225].

In addition to the NEDD4.2-ENaC axis, NEDD4.2 knockout mice revealed that the abundance of NCC on DCT cells was also increased in the absence of NEDD4.2, meanwhile the phosphorylation at T53, T58, and S71 of NCC were also increased [226]. This suggests that a parallel NEDD4.2-NCC axis can also contribute to the electrostatic balancing of ion reabsorption. Overall, it leaves an impression that preserving NEDD4.2 function is preferable to less sodium reabsorption and lower blood pressure; extending from this point, it is desirable for hypertension therapists to develop and deploy small molecules in NEDD4.2 function preservation.

### 6.2. Epithelial Na^+^ Channel (ENaC)

The epithelial sodium channel (ENaC) is a renal CCD sodium reabsorption channel consisting of three transmembrane subunits (alpha, beta, and gamma). Each subunit shares approximately 30–40% sequence identity with the others and has two presumed membrane-spanning domains, a large extracellular loop, and intracellular N- and C-termini [227,228].

Aldosterone, cortisol, Ang II, and insulin are ENaC activators [229]. As cortisol and aldosterone bind to the mineralocorticoid receptors (MR) in the principal cells of the cortical collecting duct (CCD), the ligand–receptor complexes act as transcription factors that stimulate the expression of *SCNN1* (encoding ENaC) and *SGK1* (encoding SGK1) [230]. Meanwhile, SGK1 also phosphorylates α-ENaC at the C-terminus that stimulates ENaC activity [231]. Upregulated by glucocorticoids and mineralocorticoids such as cortisol and aldosterone, ENaC is the terminal effector in apparent mineralocorticoid excess (AME) syndrome. Therefore, as long as the amount of cortisol (the upstream hormone stimulating ENaC activity) fails to be controlled by being converted into the inactive cortisone by 11B-HSD2 due to the dysfunction of 11beta-hydroxylase, ENaC can be overactivated, causing severe hypertension [232]. In general, ENaC is the major effector that bridges aldosterone and cortisol stimulation to hypertension response.

Gain-of-function mutations within SCNN1, the gene encoding ENaC that causes Liddle’s Syndrome, is characterized by increased ENaC activity and results in an autosomal dominant form of salt-sensitive hypertension, hypokalemia, metabolic alkalosis, and hyporeninemia [233,234,235]. These features were first reported together in a 16-year-old female patient by Liddle et al. in 1963 (a familial renal disorder simulating primary aldosteronism but with negligible aldosterone secretion). Recently, a systematic review of reported cases of Liddle’s Syndrome conducted in 2018 revealed that hypertension was present in 92.3% of patients with Liddle’s mutation [236]. Liddle’s mutations are deletions in the C-terminal PY motif (PPPAYATL) in subunits of ENaC, and some single amino acid replacements, such as αY673A, βY618A, and γY628A, also disrupt the binding of NEDD4.2, thereby preventing NEDD4.2-dependent inhibition [237,238,239]. This results in an increase in the number of ENaC channels in the membrane, which in turn is thought to cause the hyperabsorption of Na^+^ and hypertension in patients with Liddle’s Syndrome [71].

As sodium flushes into the aldosterone-sensitive epithelial cells through hyperactive ENaC, the basolateral Na^+^-K^+^-ATPase is promoted to decrease the high sodium gradient via exchanging more Na^+^ for K^+^, thereby largely increasing intracellular [K+]. Therefore, the large K^+^ influx stimulates the activity of K^+^-secreting channels, such as ROMK, to extrude K^+^, leading to hypokalemia [240]. Then, elevated blood pressure and low serum potassium concentrations suppress the renin-angiotensin-aldosterone system, resulting in hyporeninemia in Liddle’s Syndrome [234]. Conversely, inherited loss-of-function mutations in ENaC result in PHA1 and physiologically present as severe hypotension, renal salt wasting, metabolic acidosis, and hyperkalemia [235]. 

Besides Liddle’s mutations, multiple variants of the human ENaC gene have been identified to be related to channel activity, internalization, and degradation, including SNPs, insertion, and deletion. For example, W493R mutations in the α subunit (αW493R) and L511Q mutations in the γ subunit (γL511Q) and β subunit V348M largely increase channel activity by reducing ENaC’s self-inhibiting effect, which is caused by an increased intracellular Na^+^ concentration (Table 1) [241,242]; other sites also exist where mutations are likely to increase channel activity, including sites in the transmembrane domain that are in the vicinity of the channel gate.

Besides the mutations, the open probability of ENaC, its channel activity, and its protein levels are highly regulated by PTMs such as phosphorylation, ubiquitination, palmitoylation, and protease cleavage. The cAMP-PKA/PKC pathway has also been reported in vitro to increase ENaC internalization and degradation through phosphorylation of ENaC at βT613 and γT623 via ERK or cyclin-dependent kinases (CDKs) [243,244]. The phosphorylation of the two ERK sites have been shown to facilitate the interaction of β- and γENaC with NEDD4.2, hence increasing ENaC retrieval from the plasma membrane [244,245]. Therefore, the mutation of the two phosphorylation sites (βT613A, γT623A) increases ENaC-mediated, amiloride-sensitive whole cell currents (ΔIami) and the expression of βENaC at the cell surface by reducing the rate of ENaC retrieval [244]. It still remains to be determined how the addition of a phosphate group to residue βT613 affects the binding of the PY-motif to NEDD4.2 at the molecular level. These two sites in the C-terminal regions are deleted by Liddle’s mutations, which can explain the occurrence of ENaC gain-of-function mutations (Table 1).

Protein cleavage plays an important part in ENaC maturation, cellular localization to the plasma membrane, subunit assembly, and activity. The three subunits of ENaC are transported to the plasma membrane, assembled, and get activated only after cleavage by proteases, such as furin and prostasin. Furin cleaves the extracellular domain of the ENaC α-subunit twice (after residue 181 and 204) and the γ-subunit once (after residue 138), thereby preventing the initiation of Na^+^ self-inhibition [74,246]. These furin cleavage sites in the extracellular group contain consensus motifs RXXR for furin recognition and are cleaved after the last arginine of the motif [75]. On the other hand, the natural occurrence of spliced variants with the amino acids of α-subunit that are deleted at 34–82 significantly reduced ENaC activity, resulting in a 73% lower Na^+^ current when expressed in oocytes with WT β- and γ-ENaC, and a 28% reduction in surface expression compared with WT, as shown in recent studies [247].

Moreover, other studies indicated that the co-expression of prostasin with ENaC increases both channel open probability and cleavage at the polybasic RKRK186 tract in the γ-subunit, showing that prostasin can also recognize ENaC at the RKRK motif and cleave at the K186 site, which is distal to the furin cleavage site. An ENaC mutant with a substitution of RKRK186 to QQQQ186 prevented ENaC activation, and, interestingly, prostasin kinase-dead mutant S238A could still facilitate ENaC cleavage and activation. However, the detailed mechanism is still unclear [247,248]. Protein cleavage is necessary for the N-linked glycosylation of ENaC in the ER, and glycosylation is crucial for ENaC assembly, activity regulation, and Na^+^ self-inhibition [249]. Other studies demonstrated that there are 6, 13, and 5 consensus N-glycosylation residues (N-X-S/T) in the extracellular domain of the mouse α-, β-, and γ-subunits, respectively [249]. However, the exact glycosylation sites of mature α- and γ-ENaCs in human kidneys have not yet been clarified. Furthermore, ENaC is activated by Cys-palmitoylation. The β- and γ-subunits of ENaC are palmitoylated at specific cytoplasmic cysteine residues (βC43, βC557, γC33, and γC41). Mutating these cysteine residues prevents palmitoylation and dramatically reduces channel open probability, while the surface expression and proteolytic processing of channel subunits remain unaffected [250,251,252].

Extracellular cations such as Na^+^ and Li^+^ cause the self-inhibition of ENaC through the direct binding and saturation of ENaC transporting site capacity, which plays a significant role in ENaC inhibition [253]. Though extracellular K^+^ ions have also been demonstrated to block ENaC activation, the effect was much weaker and the mechanism of K^+^ inhibition was different from that of Na^+^ inhibition and has not been clearly defined [254]. Extracellular Na^+^ inhibits ENaC by binding to an extracellular cation sensing site at the acidic cleft without interacting with other associated proteins. Na^+^ and Li^+^ inhibit ENaC in a similar way, while Li^+^ functions at a slightly slower rate [254]. Recently, there were multiple studies providing identification of possible Na^+^ binding sites. It was also shown that a polymorphism, W493R, in the WPSXXS motif of ENaC α-subunit was related to the loss of Na^+^ self-inhibition and an increased channel open probability, implying a possible cation binding site. Also, further studies of the crystal structure of ASIC1 (acid-sensing ion channels) determined that cation binding sites are located in the finger and thumb domains of ENaC, which is exactly the domain that undergoes furin or trypsin protease cleavage to prevent Na^+^ self-inhibition [255,256]. Functional groups located in the β6-β7 loop in the β-ball domain of the ENaC alpha-subunit, including the carboxyl group of αD338 and the carbonyl oxygens of αL135, αE335, and αV346, interact with cations via their negative charge. Additionally, the hydroxyl group of αS344 interacts with the bound cation through a water molecule [255]. Moreover, previous work showed that γH239 of mouse ENaC (γH233 in human) upregulates Na^+^ self-inhibition, while αH282 in mouse ENaC (αH255 in human) inhibits Na^+^ self-inhibition, these two sites are shown to be related to the pH regulation of the channel [257]. The rate of Na^+^ and H^+^ transport have synergistic effects. Increased Na^+^ absorption generates a transmembrane potential that boosts H^+^ secretion, and the ENaC current is also mediated by H^+^ concentration. The binding of H^+^ ions at αD365 attenuates the effect of ENaC Na^+^ self-inhibition, therefore increasing overall ENaC activity. αD365W could also impair the protonation of the ENaC H^+^ binding site, reversing the effect of acidification on the ENaC current. This implies the fact that pH regulates ENaC by changing the channel gating.

As ENaC-mediated sodium transport is electrogenic, the H^+^, K^+^, and Cl^−^ gradients are also influenced by the ENaC sodium current. In DCT cells, ENaC absorbs Na^+^ ions from the lumen without carrying in negative ions, thereby causing an increased negative charge in the lumen and thus generating a negative membrane potential. With the accumulative effect of ENaC transport along DCT to CCD, this electrical gradient provides a driving force for the movement of other ions, such as K^+^ or Cl^−^, to downstream parts of distal nephrons [258]. It was previously shown that ROMK and calcium-activated K^+^ (BK) channel activation via an electrogenic effect is induced by ENaC functioning, and that multiple ion channels, including NCC and NKCC2, are all affected by the electrogenic effect on ENaC activity [258]. 

Generally, WNK4 regulates NCC in a kinase-dependent manner, while its effects on ENaC and ROMK are kinase-independent [21]. The increased K^+^ concentration in the body induces hyperaldosteronism. As for repressed ENaC activity in PHAII, it was reported to result from the upstream overactivation of NCC, largely decreasing Na^+^ concentration in the lumen, and therefore reducing the function of electric-gradient-driven ENaC. This could probably explain why ENaC was shown to have an increased effect in a previous study conducted on Xenopus oocytes with a WNK4 PHAII mutant, however, this statement still requires further research confirmation [21]. Another PHAII-causing mutation is the large intron 1479–667 deletion of WNK1, which would increase WNK1 transcription, thereby increasing the intracellular levels of WNK1 and further increasing the cell surface abundance of NCC (Figure 2) [13,19].

## 7. Conclusions

WNK centers in the hub of the SPAK/OSR1 kinase network drive robust post-translational modification cascades and prime ion channels in the kidney for the balance of electrolytes together. WNK pathways were also reported as the primary responder to the fluctuation of extracellular chloride ions, neuro-endocrines (Ang II, aldosterone, and AVP), and hydrostatic signals. Proven by independent but intercorrelated studies from GWAS cohort analysis, genetic mutant animal models, and biochemical cell studies, WNK together with the SPAK/OSR1 kinase axis stand out as the upstream regulators of key renal ion channels (NCC, NKCC2, ROMK, and ENaC). Overall, this evidence accentuates the deep involvement of the dysregulated WNK network in the pathophysiology of hypertension. In addition to hypertension, a wide spectrum of electrolyte disorders, such as hyperkalemia, hyperchloremia, and metabolic acidosis, were also found to occur with dysregulation of the WNK signaling network. While this review focused on the WNK-centric pathomechanisms in hypertension, we also surveyed the small molecule inhibitors that target the WNK-SPAK/OSR1 axis in the context of hypertension. Among the small molecule inhibitors mentioned in this article, only tenapanor has undergone clinical trials for its ability to block NHE3 from the gastrointestinal tract, and the investigation for novel kinase inhibitors of the WNK signaling pathway still provides the prospect for potential therapeutic applications in the future. By revisiting the genetic characters, molecular pathways, and pharmaceutical aspects of WNK-related hypertension, we provided an in-depth report for future therapeutic inventions and precision medicine workers to engage in the study of hypertension.

## Figures and Tables

**Table 1:** PTMs and mutation sites of kinase cascade regulating renal ion channel. Abbreviations: BP, Blood pressure; m, mice; h, human; auto, autophosphorylation; pRTA, proximal renal tubular acidosis.

Proteins	Upstream Regulator (Direct)	PTM Sites	PTM Modified Motif & Domain	PTM Effect	Effect on BP^1^	Ref	Mutation Sites	Clinical Significance *	Motif & Domain	Mutation Effect	Effect on BP	Ref
	The WNK Signaling											
WNK4	SGK1 & PKA & PKC	p-S1169(m) = p-S1190(h)	COOH termini, RRXS motifs	inhibit ENaC	↓	[21,22]	S1169D		COOH termini	WNK4 gain of function; PHAII	↑	[23]
							S1169A		COOH termini	inhibit ENaC	↓	[21]
		p-S1196(m) = p-S1217(h)	COOH termini, RRXS motifs	WNK4 S332 autophosphorylation, reduces NCC inhibition	↑	[22,24]	S1196D		COOH termini	WNK4 gain of function	↑	[23]
	AKT & SGK1	p-S47(h)	NH2 termini, RRXS motifs	WNK4 S332 autophosphorylation, reduces NCC inhibition	↑	[25]						
	PKA&PKC	p-S64(h)	NH2 termini, RRXS motifs	WNK4 S332 autophosphorylation	↑	[26]						
	SGK1	p-S1800(m) = p-S1201(h)	COOH termini, RRXS motifs	activate WNK4	↑	[22]						
	autophosphorylation	p-S332(m) = p-S335(h)	activation T-loop, kinase domain	activate WNK4	↑	[27]	S335A		activation T-loop, kinase domain	WNK4 loss of function	↓	[28]
							L322F		T-loop kinase domain	WNK4 gain of function	↑	
							R1185C		CaM binding domain	WNK4 partially loss of function	↓	[29]
							D561A		acidic motif	prevent KLHL3 bind to WNK4	↑	[29,30,31]
							Q565E	Pathogenic				
							E562K	Pathogenic				
							D321A or D321K, K186D		T-loop kinase domain	WNK4 kinase dead	↓	[32]
	p38MAPK- MK pathway	p-S575(h)	near the acidic motif	activate WNK4	↑	[33]						
WNK1	SGK1 & AKT	p-T58(m) = p-Thr60(h)	proline-rich domain (PRD)	inhibit ROMK	↑	[34,35]	T58A(m)		proline-rich domain (PRD)	WNK1 loss of function	↓	[34]
							K233M		subdomain I, kinase domain	WNK1 kinase dead	↓	[36]
	auto	p-S382(h)	activation T-loop, kinase domain	activate WNK4	↑	[17]	S382A		activation T-loop, kinase domain	WNK1 kinase dead	↓	[37]
							D368A		activation T-loop, kinase domain	WNK1 kinase dead	↓	[38]
							F316A		kinase domain	inhibit NCC	↓	[27]
							HQ/AA		coiled-coil domain, HQ motif	inhibit NCC	↓	[27]
							intron 1 479–667 deletion			WNK1 overexpression, PHAII	↑	[19,39]
WNK3	auto	p-S308, p-S304	activation T-loop, kinase domain	activate WNK3	↑	[33,40]						
SPAK	WNK1, WNK3 & WNK4	T233	activation T-loop, kinase domain	activate SPAK	↑	[7]	T233E		activation T-loop, kinase domain	SPAK gain of function	↑	[41]
		S373, S387	COOH termini, S-motif	activate SPAK	↑	[7]						
							T243A		activation T-loop, kinase domain	SPAK loss of function, Gitelman’s Syndrome	↓	[28,42]
OSR1	WNK1, WNK3 & WNK4	T185	activation T-loop, kinase domain	activate OSR1	↑	[7]	T185E		activation T-loop, kinase domain	OSR1 gain of function	↑	[41]
		S325, S339	COOH termini, S-motif	activate OSR1	↑	[7]			kidney-specific inactivation: KSP-OSR1-/-	OSR1 loss of function, Bartter’s Syndrome	↓	[43]
AKT	mTORC2	p-S473	hydrophobic motif	activate WNK1	↑	[44]						
		p-T308	activation T-loop, kinase domain	activate WNK1	↑	[44]						
SGK1	mTORC2	p-S422	hydrophobic motif	inhibit ROMK	↑	[17,45]	S422D		hydrophobic motif	SGK1 gain of function	↑	[24]
		p-T256	activation T-loop, kinase domain	activate WNK1	↑	[17,45]						
							K127M		ATP-binding site in kinase domain	SGK1 kinase dead	↓	[24]
NEDD4.2	SGK1 & 14-3-3	p-S342, p-T367, p-S448	14-3-3 binding motifs	increase ENaC expression	↑	[46,47,48]						
	AKT	p-S342, p-S428	between the WW domains	increase ENaC expression	↑	[47,49]						
	PKA/PKC	p-S327, p-S221, p-T246	between the WW domains	inhibit Nedd4-2	↑	[50]						
	NEDD4.2 WW domain	n/a	HECT domain, LPxY motif	increase ENaC expression	↑	[51]						
CUL3							exon 9 deletion		NH2 termini	CUL3 loss of function, PHAIIE	↑	[52]
KLHL3	AKT, PKC & PKA	p-S433	kelch repeat region	increase WNK4 expression	↑	[53]	S433E		Kelch repeat region	KLHL3 loss of function	↑	[54]
							R528H	Pathogenic	Kelch repeat region	KLHL3 loss of function	↑	[55]
	Proximal Tubule (PT)											
NHE3	SGK1	p-S663	RRxS motifs	increase NHE3 expression	↑	[56]	S663A		RRxS motifs	NHE3 loss of function	↓	[56]
	PKA	p-S555, p-S607	RRxS motifs	inhibit NHE3 expression	↓	[56]	S607A		RRxS motif	NHE3 gain of function	↑	[57]
NBC1	PKA	p-S1026	COOH termini	increase NBC1 expression	↑	[58]						
		p-T49	NH2 termini	increase NBC1 expression	↑	[58]						
							T485S or A799V or R881C			NBC1 gain of function	pRTA	[59,60]
	Thick Ascending Tubule (TAL)											
NKCC2	SPAK & OSR1	p-S91, p-T95, p-T100, p-T105, p-T118, p-S120, p-S130	amino acid permease N-termini	NKCC2 activated	↑	[61,62,63]						
NKCC1	SPAK & OSR1	p-T203, p-T207, p-T212, p-T230	amino acid permease N-termini	NKCC1 activated	↑	[64,65,66]						
	Cortical Collecting Duct (CCD)											
ROMK	WNK4	p-S44	inward rectifier potassium region	decrease ROMK expression	↓	[67,68]	S44D		inward rectifier potassium region	ROMK gain of function	↑	[69]
	SGK1 & PKA & PKC	p-S44	inward rectifier potassium region	increase ROMK expression	↑	[70]	S44A		inward rectifier potassium region	ROMK loss of function	↓	[69]
ENaC	NEDD4.2	u-βT613, u-γT623	PY motif	inhibit ENaC	↓	[71,72]			Truncation -SCNN1B: COOH termini SCNN1C: the PY motif, the Nedd4.2 binding site	ROMK gain of function Liddle’s Syndrome	↑	[6,73]
	Furin	cleaves α-subunit twice (after residue 181 and 204)	extracellular domain	activate ENaC	↑	[74,75,76]						
		cleaves γ-subunit once (after residue 138)	extracellular domain	activate ENaC	↑	[74,75,76]						
	prostasin	cleaves γ subunit at a defined polybasic (RKRK) tract	finger domain	activate ENaC	↑	[77]						

↓ down-regulation; ↑ up-regulation. * The clinical significance of the genetic variants included in the table were referred from ClinVar database (https://www.ncbi.nlm.nih.gov/clinvar/, accessed on 27 August 2022). Only the variants that are documented as “pathogenic” are included.

## Data Availability

Not applicable.

## Abbreviations

AKT	Protein kinase B
AME	Apparent mineralocorticoid excess
AT1R	Angiotensin receptor 1
AVP	Arginine vasopressin
Ang II	Angiotensin 2
cAMP	Cyclic adenosine monophosphate
CCD	Cortical collecting duct
CDK	Cyclin-dependent kinases
CHX	Cycloheximide
CNT	Connecting tubule
CUL3	Cullin-ring ubiquitin 3
CUL3Δex9	Cullin 3 exon 9 deletion mutant
DCT	Distal convoluted tubule
DSS rat	Dahl salt sensitive rat
DUBs	Deubiquitinating enzymes
eGFR	Estimated glomerular filter rate
END1	Endothelin-1
ENaC	Epithelial sodium chloride co-transporter
ERAD	Endoplasmic reticulum (ER)-associated degradation
ERK	Extracellular signal-regulated kinases
FHHt	Familial hyperkalemic hypertension
GR	Glucocorticoid receptor
HECT domain	Homologous to the E6-AP carboxyl terminus
HTN	Hypertension
IR	Insulin receptor
KLHL3	Kelch-like protein
KS-WNK1	Kidney-specific WNK1
L-wnk1	Long-form WNK1
MAGE-D2	Melanoma-associated antigen D2
MK pathway	MAPK-activated protein kinase pathway
MR	Mineralocorticoid receptor
mTORC2	Mechanistic target of rapamycin complex 2
NBC1	Sodium bicarbonate co-transporter 1Na^+^/3HCO^3−^ transporter
NCC	Sodium chloride co-transporter
NEDD4.2	Neural precursor cell expressed developmentally down-regulated 4 ligase
NHE3	Na^+^/H^+^ exchanger 3
NHERF2	Na^+^/H^+^ exchange regulatory factor 2
NKCC2	Na^+^/K^+^/2Cl^−^ co-transporters
NMD	Nonsense mediated decay
OS9	Osteosarcoma amplified 9 protein
OSR1	Oxidative stress responsive kinase 1 (OXSR1)
PHAII	Pseudohypoaldosteronism type II
PI3K	Phosphoinositide 3-kinases
PIP2	Phosphatidylinositol 4,5-bisphosphate
PKA	Protein kinase A
PKC	Protein kinase C
PT	Proximal tubule
PTM	Post-translational modification
pRTA	Proximal renal tubular acidosis
PY motif	PPxY motif
RAAS	Renin aldosterone angiotensin system
ROMK	Renal outer medullary potassium channel
RTA	Renal tubular acidosis
SGK1	Serum/glucocorticoid regulated kinase 1
SPAK	Ste20-related proline alanine rich kinase (STK39)
TAL	Thick ascending loop
UCH-L3	Ubiquitin carboxyl-terminal hydrolase isozyme L3
UMOD	Uromodulin = Tamm horsfall protein (THP)
V2R	Vasopressin receptor 2
VAMP	Vesicle-associated membrane protein
WNK	With-no-lysine (K) kinase
[K^+^]i	Intracellular potassium concentration
ΔIami	Amiloride-sensitive whole-cell currents
